# Circulating Plasma Cells as a Minimally Invasive Adjunct to Bone Marrow Aspirates for Genetic Analysis of ER Stress and Autophagy in Multiple Myeloma: A Feasibility Study

**DOI:** 10.3390/biomedicines14040737

**Published:** 2026-03-24

**Authors:** A.-M. Joëlle Marivel, Therese M. Becker, Alexander James, Yafeng Ma, Nirupama D. Verma, Tara L. Roberts, Silvia Ling

**Affiliations:** 1 School of Medicine, Western Sydney University, Narellan Rd & Gilchrist Dr., Campbelltown, NSW 2560, Australia; 2 Ingham Institute, 1 Campbell St., Liverpool, NSW 2170, Australia; 3 Centre for CTC Diagnostics & Research, 1 Campbell St., Liverpool, NSW 2170, Australia; 4 NSW Health Pathology Integrated Service Building (ISB), Liverpool Hospital, Cnr of Elizabeth and Goulburn Street, Liverpool, NSW 2170, Australia; 5 South West Sydney Clinical School, University of New South Wales, Eastern Campus, Burnside Dr., Liverpool, NSW 2170, Australia

**Keywords:** multiple myeloma, circulating plasma cells, ER stress, *uXBP1*, *ATF6*, *LAMP2A*, droplet digital PCR

## Abstract

**Background:** Multiple myeloma (MM) is characterised by clonal expansion of plasma cells (PCs) in the bone marrow (BM). Disease assessment and monitoring typically rely on invasive, single-site procedures, such as BM biopsies (BMBs), which may inadequately capture intra- and extra-medullary spatial heterogeneity. Circulating plasma cells (CPCs), enriched from peripheral blood (PB), may represent a minimally invasive alternative or adjunct for molecular profiling. **Objectives:** This study aimed to evaluate the feasibility of using CPCs, enriched from PB, for mRNA analysis in plasma cell dyscrasia, including MM. A secondary objective was to assess whether mRNA expression levels of the endoplasmic reticulum (ER) stress sensors *X-box-binding protein* 1 (u*XBP1*) and *activating transcription factor 6* (*ATF6*), and the chaperone-mediated autophagy marker *Lysosomal-Associated Membrane Protein 2* (*LAMP2A*) by droplet digital PCR (ddPCR), were associated with resistance to the second-generation proteasome inhibitor (PI) carfilzomib (Cfz). **Methods:** Multiple myeloma (MM) cell lines (H929 and U266) and their carfilzomib-adapted derivatives were used to establish and validate droplet digital PCR (ddPCR) assays targeting ER stress (*uXBP1, ATF6*) and autophagy-related (*LAMP2A*) transcripts. Solid tumour cell lines, including serum-starved HeLa cells, served as biological controls to support assay specificity and sensitivity. Total RNA was extracted and reverse-transcribed to complementary DNA prior to analysis. Transcript levels were normalised to those of *β-actin* or *GAPDH*, as appropriate. ddPCR was performed using the BioRad QX200 system, with results reported as the normalised transcript copy number per microlitre of reaction. Matched bone marrow aspirate (BMA) and peripheral blood (PB) samples were collected at a single clinical time point from adults undergoing investigation for plasma cell dyscrasia between January 2021 and December 2023. Samples were obtained as part of standard clinical care and/or during treatment with Bortezomib (Btz) or Cfz. Mononuclear cells were isolated by density gradient centrifugation, and CD138^+^ plasma cells were enriched by fluorescence-activated cell sorting. Enrichment purity was assessed qualitatively by immunofluorescence microscopy using CD138 and CD117 markers. Samples yielding fewer than 1000 CD138^+^ plasma cells were excluded, resulting in 10 evaluable matched patient pairs. **Results:** Carfilzomib-adapted MM cell lines demonstrated reduced levels of u*XBP1*, *ATF6*, and *LAMP2A* mRNA compared to treatment-naïve cells. In matched BM and PB samples, u*XBP1* mRNA levels were consistently lower in circulating PCs than in BM-derived PCs, whereas *ATF6* mRNA levels were concordant between compartments. *LAMP2A* mRNA levels exhibited marked inter-patient heterogeneity. **Conclusions:** This study demonstrates the feasibility of using CPCs as a minimally invasive source for mRNA-based biomarker assessment and highlights ddPCR as a sensitive platform for quantifying ER stress and chaperone-mediated autophagy related transcripts in CPCs. Cfz adaptation was associated with reduced levels of u*XBP1* and *LAMP2A* mRNA in MM cell lines. Future prospective studies evaluating the clinical utility of ER stress and chaperone-mediated autophagy associated transcripts in CPCs as predictors of resistance to PI are warranted.

## 1. Introduction

Multiple myeloma (MM) is a malignancy of plasma cells (PCs) in the bone marrow. This malignancy is both genetically and spatially heterogeneous, characterised by clonal expansion of malignant PCs that are frequently distributed in a patchy manner throughout the bone marrow and may also be present in the extra-medullary tissues [1,2,3]. Bone marrow biopsies (BMBs) are gold-standard diagnostic procedures in haematological neoplasms, including MM. In conjunction with full blood counts, measurement of monoclonal M protein levels in serum and urine by protein electrophoresis, immunofixation, serum free light chain assays, and imaging modalities such as magnetic resonance imaging (MRI), X-ray and computerised tomography (CT) scanning, BMBs are used for the diagnosis and monitoring of MM [4]. However, BMBs are single-site sampling procedures and therefore may not accurately predict the total tumour burden or capture the full spectrum of clonal heterogeneity inherent to MM [2,5]. Additionally, their invasive nature can result in significant discomfort, stress and anxiety. Circulating plasma cells (CPCs) have been observed in particularly advanced-stage MM [6]. These CPCs can be easily collected through a minimally invasive peripheral blood draw which can be performed repeatedly over the course of the disease. The utility of CPCs as biomarkers for prognosis [7,8], early relapse [9] and treatment response monitoring [10] has been investigated, although CPC analysis is not routinely implemented in clinical diagnostics. To date, CPC analysis has largely relied on flow cytometry, while the feasibility and utility of CPCs for mRNA analysis in MM remain unclear. Droplet digital PCR (ddPCR) allows absolute quantification of mRNA from very small cell numbers, making this platform suitable for transcriptomic investigations in CPCs.

In MM, there is an unmet need for minimally invasive methods to sample clonal PCs for disease progression and response assessments and early relapse detection. Despite achieving initial responses to therapy, MM is characterised by inevitable relapse with progressively shorter durations of remission following successive lines of treatment until patients ultimately develop refractory disease [11]. Consequently, the ability to predict treatment resistance, or early relapse, would facilitate more effective treatment planning and potentially improve patient outcomes.

Endoplasmic reticulum (ER) stress-adaptation pathways are central to plasma cell survival and have been implicated in resistance to PI therapy in MM. Key ER stress sensors, including *unspliced X-box-binding protein 1* (*uXBP1*) and *activating transcription factor 6* (*ATF6*) [12,13], together with a chaperone-mediated autophagy marker (*LAMP2A*), have been linked to PI resistance [14,15,16,17,18,19]. As PI-based regimes form the backbone of MM treatment, the development of refractoriness following prolonged PI exposure remains a major driver of disease relapse. Therefore, there is a critical need for robust biomarkers capable of predicting resistance, enabling decisions to avoid ineffective therapy and improve clinical outcomes.

The prognostic relevance of ER stress sensor expression has previously been explored in MM. Reduced mRNA expression of *XBP1* and *ATF6* has been reported in patients treated with the immunomodulatory agent thalidomide [20], as well as in patients resistant to the first-generation PI, Btz [15,17]. These findings support a mechanistic link between attenuation of ER stress signalling and PI resistance. However, whether *XBP1* and *ATF6* expression predict response to the second-generation PI Cfz, which displays distinct pharmacodynamics and resistance mechanisms, is currently unknown.

In this feasibility study, we sought to address two key questions. First, we evaluated the use of CPCs as a minimally invasive alternative to serial bone marrow aspirates for mRNA-based biomarker analysis. Second, we investigated whether *uXBP1*, *ATF6,* and *LAMP2A* mRNA levels are associated with resistance to Cfz in MM cell line models. To our knowledge, this is the first study to assess ER stress- and autophagy-related circulating blood biomarkers as predictors of Cfz resistance in MM.

## 2. Materials and Methods

### 2.1. Study Cohort

Matched BM aspirates (BMAs) and PB samples were collected at a single clinical time point from 13 patients under investigation for plasma cell dyscrasia. The inclusion criteria were adults who underwent BMB and PB collection for the investigation of suspected plasma cell dyscrasia as per the standard of care, and/or were treated with Btz and Cfz, during the time period from January 2021 to December 2023. Clinical data, including age at diagnosis, sex, disease status at sample collection, and progression-free survival, were obtained from the patient medical records. Only patients with sufficient matched BMA and PB for CD138^+^ PC enrichment were included. Samples yielding fewer than 1000 CD138^+^ PCs following enrichment were excluded from downstream analyses to ensure adequate RNA quantity and reliable ddPCR quantification. Following these criteria, 10 patients were eligible for ddPCR-based mRNA analyses.

### 2.2. CD138^+^ Cell Enrichment by Flow Cytometry-Based Cell Sorting

Mononuclear cells were isolated from matched BMA and PB samples by density gradient centrifugation using SepMate^TM^ tubes (STEMCELL Technologies, Tullamarine, VIC, Australia). Cells were incubated in the dark with anti-CD138-APC (Clone 359103) (Thermo Fisher Scientific, Sydney, NSW, Australia) at 4 °C for 30 min. Following washing, CD138^+^ PCs were isolated by fluorescence-activated cell sorting using a BD FACSMelody™ Cell Sorter (BD Biosciences, Sydney, NSW, Australia).

Cells were initially gated on forward scatter (FSC) and side scatter (SSC) to exclude debris, followed by doublet discrimination using SSC-H vs. SSC-A. Singlet populations were subsequently gated for CD138 expression, with unstained controls used to define CD138^+^ and CD138^−^ populations. CD138^+^ cells were collected for downstream analyses. Representative flow cytometry gating strategies for BMA- and PB-derived samples are shown in the Supplementary Materials.

It should be noted that the enrichment strategy employed prioritised maximal recovery of viable cells for downstream mRNA analysis. While broader marker panels can improve specificity in conventional immunophenotyping, the use of CD138 alone enabled a balance among cell yield, viability, cost effectiveness and compatibility with immunofluorescence and ddPCR-based transcript analysis. Accordingly, this enrichment approach was feasibility-focused.

Post-sort purity was assessed by immunofluorescence microscopy.

### 2.3. Immunofluorescence Microscopy

Volumes of 200 µL of the enriched CD138^+^ MM cell suspensions and CD138^−^ cell suspensions were centrifuged onto glass slides using a CytoSpin™ system (Thermo Fisher Scientific, Sydney, NSW, Australia) at 400× *g* for 5 min. Slides were air-dried overnight at room temperature and fixed with 4% paraformaldehyde for 10 min, followed by two phosphate-buffered saline (PBS) washes. Cells were blocked overnight at 4 °C with blocking buffer containing 5% foetal calf serum (FCS) and 0.1% Triton X100 in PBS. After blocking, the slides were washed with PBS once. Slides were then incubated for 90 min at room temperature with primary antibodies diluted in 1% FCS/PBS: purified rabbit anti-human CD117 (clone 104D2) and purified mouse anti-human CD138 (clone MI15, IgG1 isotype; both from BD Biosciences, Sydney, NSW, Australia). CD138 is a hallmark plasma cell marker, while CD117 is associated with malignant plasma cells.

After three PBS washes, slides were incubated in the dark for 60 min at room temperature with secondary antibodies (anti-mouse Alexa Fluor^®^ 555 and anti-rabbit Alexa Fluor^®^ 488 in 1% FCS in PBS; Thermo Fisher Scientific, Sydney, NSW, Australia). Following three additional PBS washes, slides were mounted using 5 µL of Prolong Gold (Thermo Fisher Scientific, Sydney, NSW, Australia), cured overnight in the dark, and sealed the following day. Immunofluorescence analyses were qualitative and used to assess marker localisation rather than to quantify enrichment purity.

### 2.4. Cell Lines and Culture Conditions

The H929 and U266 MM cell lines were kindly provided by Professor Andrew Spencer (Monash Health, Melbourne, VIC, Australia). The HeLa cells were kindly provided by Associate Professor Tara L. Roberts (Western Sydney University, Campbelltown, NSW, Australia). Solid tumour cells (LNCaP, CRC SW48) were kindly provided by Associate Professor Keiran Scott (Western Sydney University, Campbelltown, NSW, Australia).

H929 and U266 MM cell lines were cultured and adapted to increasing concentrations of Cfz. The parent cell lines were named H929S and U266S. The Cfz-adapted counterparts were named H929R and U266R respectively. These cell lines were used to establish three ddPCR assays and assess the level of ER stress (u*XBP1* and *ATF6*) and chaperone-mediated autophagy (*LAMP2A*). Solid tumour cell lines LNCaP, CRC SW48, and HeLa were used to validate assay performance. Briefly, the cells were cultured in complete growth medium with 90% RPMI media (Sigma Aldrich (Merck), Bayswater, VIC, Australia), 10% foetal calf serum (FCS) and 0.01% penicillin/streptomycin/glutamate (PenStrepGlu; Sigma Aldrich (Merck), Australia) in a humidified incubator at 37 °C with 5% CO_2_ atmosphere. Serum-starved conditions were generated by omission of FCS and served as a positive control for the induction of ER stress and autophagy in HeLa cells [21].

### 2.5. RNA Extraction and cDNA Synthesis

Total RNA was extracted using Tri-Reagent (Sigma-Aldrich, Australia) according to the manufacturer’s protocol. Complementary DNA (cDNA) synthesis was performed using a Bioline reverse transcription kit (Meridian Bioscience, Eveleigh, NSW, Australia) according to the manufacturer’s instructions. Reactions were prepared on ice, in a final volume of 20 µL with 10 µL of total RNA, 4 µL of 5× TransAmp Buffer, 1 µL of reverse transcriptase and 5 µL of nuclease-free water. Reverse transcription was performed under the following conditions: 25 °C for 10 min, 42 °C for 15 min, and 85 °C for 5 min, followed by a 4 °C hold.

### 2.6. Droplet Digital PCR (ddPCR)

Custom ddPCR assays were designed for u*XBP1*, *ATF6*, and *LAMP2A* cDNA. u*XBP1* and *ATF6* were normalised to *β-actin*, while *LAMP2A* was normalised to *GAPDH*. Primers and probes were designed using the PrimerQuest™ Tool from (Integrated DNA Technologies, (IDT, Singapore) and synthesised by IDT (Singapore)). Oligonucleotides were resuspended in nuclease-free water and stored at −20 °C. Probe and primer sequences are provided in Table 1.

Assays were established using MM cell lines (treatment-naïve H929S and U266S; Cfz-adapted H929R and U266R). Solid tumour cell lines (LNCaP, SW48, HeLa, and serum-starved HeLa) served as biological controls to support assay specificity and sensitivity. Serum starvation on HeLa cells acts as a well-established positive control for the induction of ER stress and autophagy [21].

ddPCR reactions were performed on the QX200™ Droplet Digital PCR System (Bio-Rad, Granville, NSW, Australia). Each 22 µL reaction consisted of 1× ddPCR Supermix for Probes (no dUTP; Bio-Rad, Granville, NSW, Australia), 0.5 µM primers, 0.125 µM hydrolysis probe, and 5 µL cDNA. Droplets were generated using the QX100™ Droplet Generator (Bio-Rad, Granville, NSW, Australia) and transferred to semi-skirted 96-well plates (Eppendorf, Australia), which were sealed with pierceable foil seals (Bio-Rad, Granville, NSW, Australia).

Thermal cycling was performed on a C1000 Touch™ thermal cycler (Bio-Rad, Granville, NSW, Australia) under the following conditions: 95 °C for 10 min; 40 cycles of 94 °C for 30 s and 56 °C for 60 s; and a final enzyme deactivation step at 98 °C for 10 min. Droplets were analysed using the QX200™ Droplet Reader (Bio-Rad, Granville, NSW, Australia), and data were processed with QuantaSoft™ Analysis Pro software (version 1.7.4, (Bio-Rad, Granville, NSW, Australia)). Thresholds were manually set according to distinct positive and negative droplet populations for each assay.

### 2.7. Statistical Analysis

Statistical analyses were performed using GraphPad Prism (version 11.0.0 (93); GraphPad Software, Boston, MA, USA). Given the exploratory nature of this study and the limited sample size, analyses were primarily descriptive.

One-tailed unpaired *t*-tests were used for cell line comparisons based on a priori hypotheses of reduced gene expression in Cfz-adapted cells and increased ER stress and autophagy-related gene expression under serum starvation.

Paired comparisons between bone marrow-derived and circulating CD138^+^ plasma cells were assessed using Wilcoxon matched-pair signed-rank tests. Normality was assessed using the Shapiro–Wilk test.

For comparisons between two independent groups, including circulating plasma cells (CPCs) and resident bone marrow (BM) plasma cells, the Mann–Whitney U test was applied due to the non-normal data distribution and sample size considerations. Differences were considered statistically significant at a two-sided *p* value < 0.05.

ddPCR results are reported as normalised transcript copy numbers per microlitre of reaction.

## 3. Results

### 3.1. Patient Cohort

Matched BMA and PB were collected from 13 patients under investigation for plasma cell dyscrasia (Table 2). Twelve patients were used for cell enrichment as patient MM020 was subsequently not diagnosed with MM. Following CD138^+^ cell enrichment, sufficient plasma cell yield for mRNA analysis was obtained from 10 paired samples, which were included in downstream ddPCR analyses.

The cohort included patients spanning the disease spectrum, including monoclonal gammopathy of undetermined significance (MGUS), smouldering MM (SMM), newly diagnosed MM (NDMM), and post autologous stem cell transplantation (ASCT). Due to the small sample size and the exploratory nature of this feasibility study, analyses were descriptive, and findings were interpreted qualitatively, with emphasis on expression trends rather than formal inferential comparisons.

### 3.2. ddPCR Assay Design and Validation

**Figure 1:**
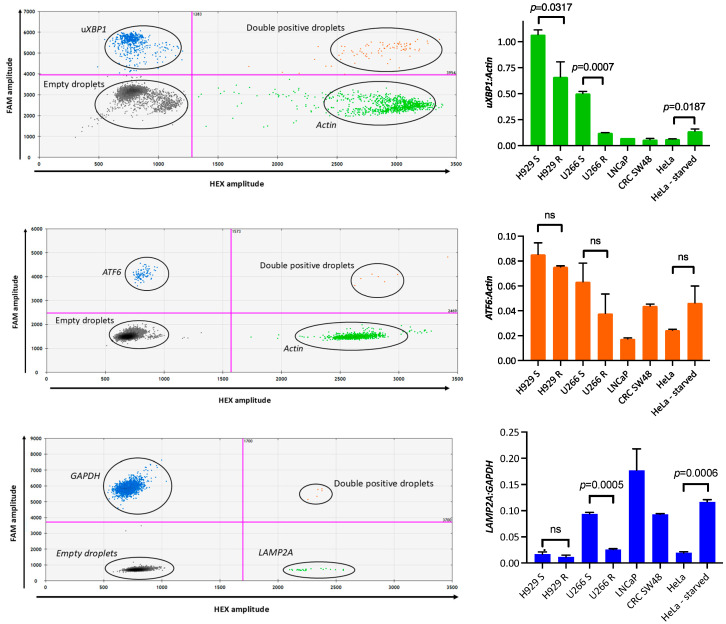
mRNA levels of u*XBP1*, *ATF6* and *LAMP2A* in cell line models. Total RNA extracted from multiple myeloma (MM) and solid tumour cell lines was reverse-transcribed to cDNA and analysed by droplet digital PCR (ddPCR). (**Left**): Representative two-dimensional ddPCR amplitude plots for *uXBP1*, *ATF6*, and *LAMP2A* demonstrating clear separation between positive and negative droplet populations for each assay. (**Right**): Normalised mRNA expression of *uXBP1* and *ATF6* (normalised to *β-actin*) and *LAMP2A* (normalised to *GAPDH*). Treatment-naïve MM cell lines (H929S, U266S) and Cfz-adapted MM cell lines (H929R, U266R) are shown alongside control solid tumour cell lines (LNCaP, SW48, and HeLa cells). HeLa cells were cultured under either complete-medium or serum-starved conditions, which served as positive controls for induction of ER stress and autophagy [21]. Bars represent mean; error bars indicate range of two technical replicates; ns: not significant.

Custom droplet digital PCR (ddPCR) assays were successfully designed and validated for the detection of *uXBP1*, *ATF6*, and *LAMP2A* transcripts. Representative two-dimensional amplification plots demonstrated clear and consistent separation between positive and negative droplet populations for each target and reference gene, indicating robust assay performance (Figure 1, left panel).

Normalised mRNA expression levels for *uXBP1*, *ATF6*, and *LAMP2A* across MM and solid tumour cell line models are shown in Figure 1 (right panels). All assays yielded reproducible quantification across technical replicates, supporting their suitability for downstream analyses in both BM-derived PCs and CPCs.

Comparisons between parental (sensitive) and Cfz-adapted MM cell lines were performed using an unpaired *t*-test with a one-tailed significance threshold (Figure 1, right panel). In the H929 cell line pair, *uXBP1* mRNA expression was significantly reduced in Cfz-adapted cells compared with parental cells (*p* = 0.0317), whereas no significant difference was observed for *ATF6* (*p* = 0.1324) or *LAMP2A* (*p* = 0.1472). In the U266 cell line pair, *uXBP1* (*p* = 0.0007) and *LAMP2A* (*p* = 0.0005) mRNA expression levels were significantly reduced in Cfz-adapted cells, while *ATF6* expression did not differ significantly between parental and adapted cells (*p* = 0.1178).

Comparisons between HeLa cells cultured under complete-medium and serum-starved conditions were performed using an unpaired one-tailed *t* test. Serum starvation was associated with a significant increase in *uXBP1* and *LAMP2A* mRNA expression (*p* = 0.0187 and *p* = 0.0006, respectively), whereas no statistically significant change was observed in *ATF6* expression (*p* = 0.0734).

### 3.3. Comparison of Circulating PCs vs. Bone Marrow PCs

To evaluate the feasibility of enriching plasma cells from both BMA and PB, CD138^+^ cell isolation was performed on 13 matched BM and PB samples (Table 2).

The frequency of CD138^+^ cells within the singlet mononuclear cellular population ranged from 0.52 to 34.5% in BMA samples and from 0.18 to 4.32% in PB samples (Supplementary Material). Immunofluorescence microscopy using CD117 and CD138 co-staining confirmed successful enrichment of CD138^+^ malignant plasma cells (Supplementary Materials).

To assess the relative yield of plasma cells from PB compared with matched BMA samples, the absolute number of CD138^+^ cells recovered per sample was compared (Table 2). Across the cohort, the number of CD138^+^ cells obtained from BMA ranged from 751 to 1.2 × 10^6^, while PB yields ranged from 299 to 1.6 × 10^5^ cells.

Although BMA generally yielded higher numbers of CD138^+^ cells, in several patients the PB samples yielded comparable or greater numbers than their matched BM counterparts. Notably, successful enrichment of CD138^+^ plasma cells from PB was achieved even in patients with low plasma cell percentages (<10%) reported in their diagnostic bone marrow pathology.

**Figure 2:**
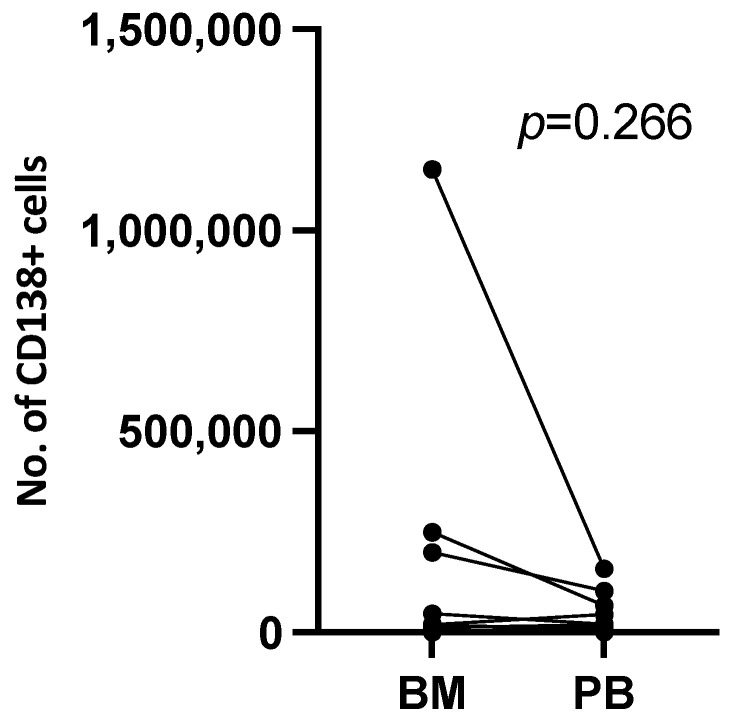
Yield of CD138^+^ plasma cells from matched bone marrow and peripheral blood samples. Absolute numbers of CD138^+^ cells enriched from bone marrow and peripheral blood were compared. Paired analysis using Wilcoxon matched-pairs signed-rank test showed no significant difference between compartments (*p* = 0.266).

Paired comparison of CD138^+^ cell yields from matched BM and PB samples using a Wilcoxon matched-pairs signed-rank test did not reveal a statistically significant difference between compartments (*p* = 0.266; Figure 2). While underpowered to detect small differences, these findings support the technical feasibility of reproducible CD138^+^ plasma cell enrichment from peripheral blood in patients across disease stages.

### 3.4. Comparison of uXBP1, ATF6, and LAMP2A mRNA Expression in Bone Marrow-Derived and Circulating Plasma Cells

**Figure 3:**
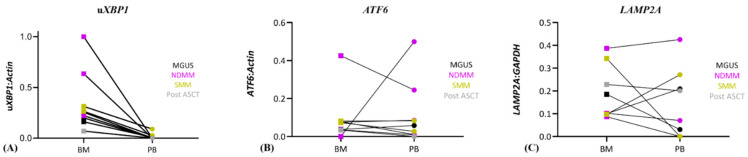
Normalised mRNA levels in matched patient samples. Samples were grouped by disease status as indicated. Outliers (samples with results only from either BM or PB samples and samples with less than 1000 CD138^+^ enriched cells) were removed from final analysis. (**A**) u*XBP1*; (**B**) *ATF6*; (**C**) *LAMP2A*. mRNA levels of u*XBP1* and *ATF6* were normalised to mRNA level of *β-actin*, while mRNA level of *LAMP2A* was normalised to mRNA level of *GAPDH*. MGUS: monoclonal gammopathy of undetermined significance; NDMM: newly diagnosed MM; SMM: smouldering MM; Post ASCT: post autologous stem cell transplant.

The second objective was to determine whether CD138^+^ PCs enriched from PB could be used for mRNA-based biomarker analysis. To address this, mRNA levels of u*XBP1*, *ATF6* and *LAMP2A* were compared between CD138^+^ cells enriched from matched BMA and PB samples (Figure 3).

Paired samples with insufficient cell numbers or RNA quantities from either compartment were excluded, leaving a final dataset of 10 matched samples for analysis.

Across the paired samples, *uXBP1* mRNA levels were consistently lower in circulating plasma cells compared with BM-derived plasma cells (Mann–Whitney U test, *p* = 0.0018). In contrast, *ATF6* mRNA levels showed substantial concordance between BM and PB compartments, except for one sample, with no uniform directional difference observed (Mann–Whitney U test, *p* = 0.69). *LAMP2A* mRNA levels displayed marked inter-patient heterogeneity, with variable differences between BM and PB from individual patients.

These findings indicate that CPCs capture transcriptional features relevant to ER stress signalling, while also revealing biologically plausible compartment-specific differences, particularly for *uXBP1*. The variability observed in *LAMP2A* mRNA levels underscores the complexity of autophagy-related regulation in clinical samples and highlights the need for larger, prospective studies to define clinically meaningful thresholds.

## 4. Discussion

In this prospective, single-centre feasibility study, we evaluated the potential of CPCs as a minimally invasive alternative or adjunct to BMB for mRNA-based biomarker assessment in plasma cell dyscrasia, including MM. We further explored the feasibility of selected ER stress and chaperone-mediated autophagy-related transcripts—*uXBP1*, *ATF6*, and *LAMP2A*—as candidate biomarkers of resistance to the second-generation PI Cfz.

Our results address an important unmet need in MM. Current diagnostic and monitoring strategies rely heavily on BMA and BMB, which are associated with mild procedural risk and significant discomfort for the patients. These procedures are performed at a single anatomical site and may fail to capture the spatial heterogeneity characteristic of MM bone marrow involvement. Furthermore, the quality of BMA and BMB can be affected by technical factors such as bone marrow infiltrative patterns, haemodilution and clot formation, potentially leading to under-representation of clonal plasma cells.

Clonal PCs acquire the capacity to egress from the BM niche, circulate in the bloodstream, and seed extra-medullary sites—features usually associated with adverse prognosis [22,23]. CPCs are therefore likely to represent biologically relevant disease-associated cells. Using BM-derived PCs as a reference, we demonstrated that enrichment of CD138^+^ PCs from PB (CPCs) yields sufficient material for downstream mRNA analysis across a spectrum of disease states, including in MGUS patients with low marrow PC burden. Notably, in several cases the CPC yield was comparable to or higher than the yield from matched BMA samples, supporting the potential utility of CPCs when marrow sampling may miss focal disease involvement.

From a translational perspective, CPCs combined with ddPCR-based mRNA quantification represents a technically feasible approach that could be implemented in diagnostic laboratories. However, clinical translation would require access to flow cytometric sorting and ddPCR platforms, standardisation of enrichment protocols, and rigorous inter-laboratory validation to ensure reproducibility.

The second objective of this study was to assess whether CPCs could be used for mRNA-based biomarker analysis relevant to PI resistance. We focused on *XBP1* and *ATF6*, two key ER stress sensors, and *LAMP2A,* a chaperone-mediated autophagy marker. Our laboratory has previously implicated reduced levels of *XBP1* and *ATF6* mRNA and increased levels of *LAMP2A* mRNA in resistance to the first-generation PI, Btz [15,18,19]. Here, we extended those observations by demonstrating, for the first time, that *uXBP1* mRNA levels were reduced in Cfz-adapted MM cell lines. While comprehensive gene panel testing has become more accessible, in the context of proteasome inhibition in MM, a small gene panel is cost-effective and allows for repeat evaluation as the disease biology evolves over time.

Validation of our ddPCR assays in solid tumour cell line models confirmed biologically appropriate induction of ER stress and chaperone-mediated autophagy under serum starvation conditions, consistent with the established stress response. In particular, increased u*XBP1* mRNA levels in serum-starved HeLa cells supported assay sensitivity to ER stress activation. The marked increase in *LAMP2A* mRNA levels under starvation further reflected engagement of chaperone-mediated autophagy, which is known to interact with ER homeostasis [24].

When applied to matched BMA and PB samples, all three transcripts were detectable in CPCs when CD138^+^ yields exceeded 1000. However, u*XBP1* mRNA levels were consistently lower or undetectable in CPCs compared to BM-derived PCs. This observation suggests that transcriptional profiles of PCs may change upon entry into the circulation. Indeed, CPCs are known to exhibit altered phenotypic and functional characteristics, including downregulation of adhesion molecules and integrins, and a loss of or reduction in CD138 expression itself has been described during plasma cell trafficking [25,26,27]. Such phenotypic adaptations may underlie the compartment-specific differences observed in *uXBP1* mRNA levels.

*LAMP2A* mRNA levels were variable across individuals in both CPCs and bone marrow PCs. It is possible that genotypic and phenotypic adaptations enabling PCs to enter the circulation influence *LAMP2A* mRNA levels.

This study demonstrated concordance of *ATF6* mRNA levels between BM and PB compartments. This raised the possibility that *ATF6* mRNA measurement in CPCs could serve as a practical surrogate for BM-derived analysis, although clinical relevance will need validation in larger, longitudinal cohorts including relapsing/refractory MM patients.

Using this assay, we demonstrated for the first time in MM cell line models that long-term adaptation to Cfz is associated with downregulation of the key ER stress regulator, *uXBP1*. The downregulation of *uXBP1* observed in Cfz-adapted cells aligned with our previous observations in Btz-resistant models [15]. In contrast, previous work from our laboratory demonstrated that long-term adaptation to Btz resulted in upregulation of *LAMP2A* [17,19]. In the present study, however, long-term Cfz adaptation did not upregulate *LAMP2A* expression and may instead have reduced *LAMP2A* expression in at least one cell line model. These divergent adaptive responses might reflect differences in the pharmacodynamic properties of Cfz and Btz [28], as well as intrinsic phenotypic differences between MM cell lines. However, this interpretation remains speculative, and the observed findings require further investigation and independent validation.

*ATF6* mRNA expression appeared to be consistent across PB and BM compartments. While prior studies identified reduced *ATF6* mRNA levels as a biomarker of resistance to Btz in both MM cell lines and patients, further investigations into the predictive value of CPC *ATF6* mRNA expression in Btz resistance are warranted. In the present study, no significant difference was found in *ATF6* mRNA levels between sensitive and Cfz-adapted MM cell line models, likely due to the limited sample size or a unique biological feature of Cfz resistance.

This study had several limitations that warrant consideration. Firstly, mechanistic and expression analyses were primarily conducted in MM cell line models adapted to Cfz. Validation with knockdown and overexpression studies, mechanistic perturbation of autophagy pathways, cross-comparison with other PIs, and evaluation in primary human samples were beyond the scope of this work. Additionally, correlation of these findings with clinical responses to Cfz will be required to confirm the underlying mechanism of resistance and establish their clinical significance. Secondly, the lack of longitudinal sampling precluded assessment of dynamic changes in transcript levels over the course of treatment. Thirdly, the small cohort size limited the statistical power for biomarker discovery. Accordingly, our findings should be interpreted as exploratory and hypothesis-generating rather than predictive of clinical outcomes.

## 5. Conclusions

Our findings support the use of CD138^+^ CPCs as a non-invasive adjunct to BMA and demonstrate ddPCR as a sensitive platform for biomarker detection in both BM-derived PCs and CPCs. It is important to note that gene expression patterns may differ between BM-derived plasma cells and CPCs, reflecting variations in microenvironmental influences and underlying biological states. Prospective longitudinal validation in larger MM cohorts treated with Cfz will be necessary to establish the clinical utility of ER stress and chaperone-mediated autophagyassociated transcripts in CPCs as predictors of PI resistance.

## Figures and Tables

**Table 1:** List of primers and probes.

Target Genes			
	u*XBP1*	*ATF6*	*LAMP2A*
**Forward primer**	5′-TGTAGTTGAGAACCAGGAGTTAAG-3′	5′-GGGTTAGAGGCGAGATTAAAGG-3′	5′-GACAACTTCCTTGTGCCCATA-3′
**Reverse primer**	5′-CCACTGGCCTCACTTCATT-3′	5′-TCTGGTTCTCTGACACAACTTC-3′	5′-CAGCATGATGGTGCTTGAGA-3′
**Taqman Probe**	5′-[56FAM]-TCTTCAGCAACCAGGGCATCCATC-[3BHQ_1]-3′	5′-[56FAM]-AAATGGAACACTGAAGCGGCAGC-[3BHQ_1]-3′	5′-[5Hex]-AGCTGCCTTGGCAGGAGTACTTAT-[3BHQ_1]-3′
Reference Genes			
	*β-actin*	*GAPDH*	
**Forward primer**	5′-CAGGTCATCACCATTGGCAA-3′	5′-CGGGAAGCTTGTCATCAATGG-3′	
**Reverse primer**	5′-AGGTAGTTTCGTGGATGCCA-3′	5′-CTCCACGACGTACTCAGCG-3′	
**Taqman Probe**	5′-[5HEX]-AGGACTCCATGCCCAGGAA-[3BHQ_1]-3′	5′-[56-FAM]-TCTTCCAGGAGCGAGATCCCT-[3BHQ_1]-3′	

**Table 2:** Patients with matched BM and PB samples.

Sample	Age at Diagnosis	Sex	PFS * (Months)	% Plasma Cells in Pathology Reports		Numberof Cells After Enrichment		Disease Status at Collection
				Bone Marrow Aspirate	Bone Marrow Aspirate (Trephine)	BM	PB	
MM004	85	F	41; no progression, alive	1	3	47,292	20,836	MGUS
MM005	86	M	41; no progression, alive	5	5–10	199,586	103,531	MGUS
MM019	70	M	lost to follow up	1	2	751	1887	MGUS
MM017	85	M	21; no progression, alive	7	15	4238	3217	SMM
MM010	67	M	14; no progression but deceased	3	10	21,170	299	SMM
MM011	66	M	40; no progression, alive	3	10–15	4464	6802	SMM
MM001	61	F	41; progression, alive	2	80	19,758	43,884	NDMM
MM007	76	M	11; progression, deceased	67	90	249,809	66,780	NDMM
MM008	84	M	16; progression, alive	13	60–70	1,152,000	158,365	NDMM
MM012	54	F	40; no progression, alive	44	80–90	16,506	13,182	NDMM
MM016	76	F	38; no progression, alive	23	not evaluable	1986	6237	NDMM
MM013	45	M	40; no progression, alive	3	5	11,387	19,407	D + 100 ASCT

* PFS was calculated from the date of diagnosis (except for MM013; date of sample collection) to censor date 23 July 2024 or the date of progression, whichever occurred first. MGUS: pre-malignant-stage monoclonal gammopathy of undetermined significance. NDMM: newly diagnosed MM. Post ASCT: autologous stem cell transplant. SMM: smouldering MM. PFS: progression-free survival.

## Data Availability

The original contributions presented in this study are included in the article/Supplementary Material. Further inquiries can be directed to the corresponding author.

## Supplementary Materials

The following supporting information can be downloaded at: https://www.mdpi.com/article/10.3390/biomedicines14040737/s1, Table S1: Data from the BD FACS Melody cell sorter. The percentage of CD138^+^ cells within the total cell population isolated by density-gradient centrifugation using SepMate tubes. Figure S1: Flow cytometric enrichment and validation of CD138^+^ plasma cells from BM and PB. (A,B) Representative FACS gating strategies for CD138^+^ plasma cell enrichment from BM and PB mononuclear cells. Cells were gated on FSC/SSC, subjected to doublet exclusion, and classified as CD138^+^ or CD138^−^ using unstained controls. (C) Representative immunofluorescence images of enriched CD138^+^ cells stained for CD117 (orange) and CD138 (purple); nuclei stained with DAPI (blue). Images shown are representative; poor nuclear morphology was due to cytospin preparation artifacts.

## Abbreviations

The following abbreviations are used in this manuscript:

ATF6	*Activating transcription factor 6*
BM	Bone marrow
BMA	Bone marrow aspirate
BMB	Bone marrow biopsy
Btz	Bortezomib
Cfz	Carfilzomib
ER	Endoplasmic reticulum
LAMP2A	*Lysosomal-Associated Membrane Protein 2*
MM	Multiple myeloma
PB	Peripheral blood
PC	Plasma cell
uXBP1	Unspliced *X-box-binding protein* 1
