# Supplementary material for: Circulating Plasma Cells as a Minimally Invasive Adjunct to Bone Marrow Aspirates for Genetic Analysis of ER Stress and Autophagy in Multiple Myeloma: A Feasibility Study
Source: Biomedicines. 2026 Mar 24;14(4):737. doi: 10.3390/biomedicines14040737 (PMC13113425; doi:10.3390/biomedicines14040737)
Supplement: Supplementary file 1 [file biomedicines-14-00737-s001.zip › biomedicines-4068349-supplementary.pdf]

Supplementary Materials:

Supplementary Table S1. Data from the BD FACS Melody cell sorter. The percentage of CD138^{+} cells within the total cell population isolated by density-gradient centrifugation using SepMate tubes.

Patient ID	Sample ID	Freq. of Parent	Cells | Freq. of Parent	Cells/Single Cells | Freq. of Parent	Cells/Single Cells/CD138^{+} | Freq. of Parent	Cells/Single Cells/CD138^{-} | Freq. of Parent
MM001	BM001	100	41.6	97.3	1.62	98.3
MM001	PB001	100	84.1	98.2	0.61	99.4
MM004	BM004	100	52.4	95.2	0.52	99.4
MM004	PB004	100	83.3	94.2	1.58	98.2
MM005	BM005	100	44.6	96.6	1.26	98.6
MM005	PB005	100	78.9	94.9	0.18	99.7
MM007	BM007	100	77.7	92.2	11.1	88.2
MM007	PB007	100	89.2	97.5	0.25	99.7
MM008	BM008	100	68.4	91.4	34.5	64.7
MM008	PB008	100	77.6	95.4	3.45	96.4
MM009	BM009	100	17.1	98	3.35	96.4
MM009	PB009	100	89.2	97	3.12	96.9
MM010	BM010	100	36.1	94.5	1.69	98.1
MM010	PB010	100	54.3	90.4	1.82	98
MM011	BM011	100	47	94.3	0.67	99.2
MM011	PB011	100	59.1	96.1	2.01	97.8
MM016	BM016	100	50.9	98.2	1.96	97.8
MM016	PB016	100	71.6	95	0.72	99.2
MM017	BM017	100	24.8	97.4	0.57	99.4
MM017	PB017	100	71.4	94	1.19	98.7
MM019	BM019	100	32.5	92.2	1.4	98.2
MM019	PB019	100	52.4	91	1.85	97.8
MM020	BM020	100	39.1	88.4	5.28	94.2
MM020	PB020	100	74.4	93.5	4.32	95.2

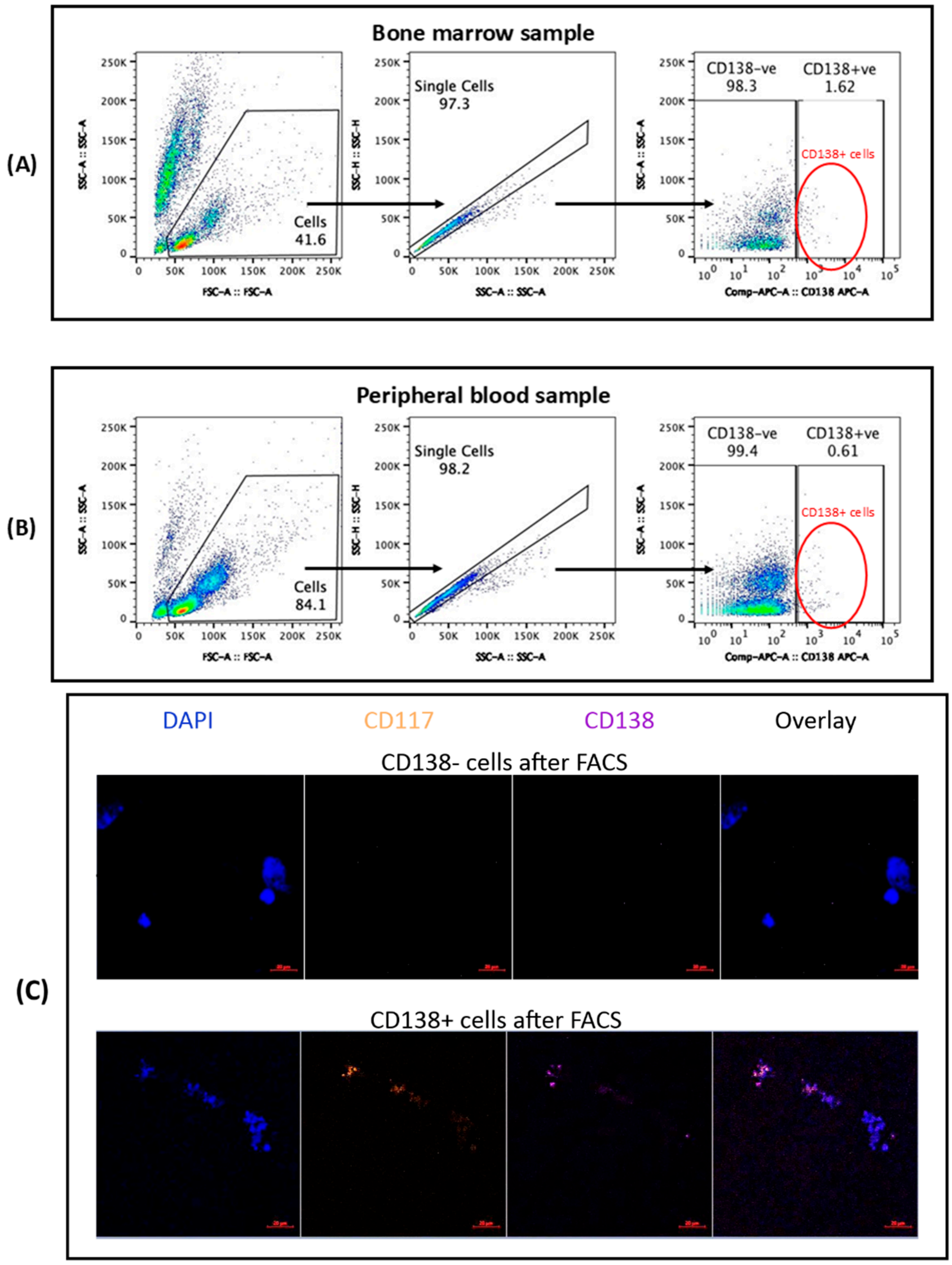

Supplementary Figure S1. Flow cytometric enrichment and validation of CD138⁺ plasma cells from BM and PB. (A-B) Representative FACS gating strategies for CD138⁺ plasma cell enrichment from BM and PB mononuclear cells. Cells were gated on FSC/SSC, subjected to doublet exclusion, and classified as CD138⁺ or CD138⁻ using unstained controls. **(C)** Representative immunofluorescence images of enriched CD138⁺ cells stained for CD117 (orange) and CD138 (purple); nuclei stained with DAPI (blue). Images shown are representative; poor nuclear morphology was due to cytospin preparation artifacts.
